# Reduction in omission events after implementing a Rapid Response System: a mortality review in a department of gastrointestinal surgery

**DOI:** 10.1186/s12913-023-09159-3

**Published:** 2023-02-21

**Authors:** Siri Lerstøl Olsen, Bjørn S Nedrebø, Kristian Strand, Eldar Søreide, Jan Terje Kvaløy, Britt Sætre Hansen

**Affiliations:** 1 Department of Quality and Health Technology, Faculty of Health Sciences, SHARE—Centre for Resilience in Healthcare, University of Stavanger, Kjell Arholms gate 43, 4036 Stavanger, Norway; 2 Department of Emergency Medicine, Stavanger University Hospital, Stavanger, Norway; 3 Department of Gastrointestinal Surgery, Haukeland University Hospital, Bergen, Norway; 4 Department of Intensive Care, Stavanger University Hospital, Stavanger, Norway; 5 Faculty of Health Sciences, University of Stavanger, Stavanger, Norway; 6 Section for Quality and Patient Safety, Stavanger University Hospital, Stavanger, Norway; 7 Department of Mathematics and Physics, Faculty of Science and Technology, University of Stavanger, Stavanger, Norway; 8 Research Department, Stavanger University Hospital, Stavanger, Norway; 9 The Research Group for Nursing and Health Care Science, Stavanger University Hospital, Stavanger, Norway

**Keywords:** Rapid Response Systems, Mortality review, Health care improvement, Improvement, Patient safety, Adverse events, Omission events.

## Abstract

**Background:**

Hospitals worldwide have implemented Rapid Response Systems (RRS) to facilitate early recognition and prompt response by trained personnel to deteriorating patients. A key concept of this system is that it should prevent ‘events of omission’, including failure to monitor patients’ vital signs, delayed detection, and treatment of deterioration and delayed transfer to an intensive care unit. Time matters when a patient deteriorates, and several in-hospital challenges may prevent the RRS from functioning adequately. Therefore, we must understand and address barriers for timely and adequate responses in cases of patient deterioration. Thus, this study aimed to investigate whether implementing (2012) and developing (2016) an RRS was associated with an overall temporal improvement and to identify needs for further improvement by studying; patient monitoring, omission event occurrences, documentation of limitation of medical treatment, unexpected death, and in-hospital- and 30-day mortality rates.

**Methods:**

We performed an interprofessional mortality review to study the trajectory of the last hospital stay of patients dying in the study wards in three time periods (P1, P2, P3) from 2010 to 2019. We used non-parametric tests to test for differences between the periods. We also studied overall temporal trends in in-hospital- and 30-day mortality rates.

**Results:**

Fewer patients experienced omission events (P1: 40%, P2: 20%, P3: 11%, P = 0.01). The number of documented complete vital sign sets, median (Q1,Q3) P1: 0 (0,0), P2: 2 (1,2), P3: 4 (3,5), P = 0.01) and intensive care consultations in the wards ( P1: 12%, P2: 30%, P3: 33%, P = 0.007) increased. Limitations of medical treatment were documented earlier (median days from admission were P1: 8, P2: 8, P3: 3, P = 0.01). In-hospital and 30-day mortality rates decreased during this decade (rate ratios 0.95 (95% CI: 0.92–0.98) and 0.97 (95% CI: 0.95–0.99)).

**Conclusion:**

The RRS implementation and development during the last decade was associated with reduced omission events, earlier documentation of limitation of medical treatments, and a temporal reduction in the in-hospital- and 30-day mortality rates in the study wards. The mortality review is a suitable method to evaluate an RRS and provide a foundation for further improvement.

**Trial registration:**

Retrospectively registered.

**Supplementary Information:**

The online version contains supplementary material available at 10.1186/s12913-023-09159-3.

## Background

Although most hospital deaths result from severe illness or injury, hospital mortality is still a quality indicator because some deaths may result from patient harm [1]. Patient harm or adverse events (AE) can be defined as “unintended injuries among hospitalised patients that result in disability, death or prolonged hospital stay, and are caused by healthcare management” [2]. The Global Trigger Tool is a commonly used tool to identify and report adverse events in hospitals [3]. Care not delivered, ‘omission events’, is found to be better detected by patient record reviews [4].

A voiced patient safety concern is the inadequate monitoring and follow-up of deteriorating patients in hospital wards [5]. Hospitals worldwide have implemented RRSs to remedy this. By concept, an RRS supports healthcare professionals in the early recognition of patient deterioration and securing prompt response by trained personnel evaluating and caring for the patient [6]. Thus, central to the RRS is to help prevent ‘omission events’, including failure to monitor the patients vital signs, delayed detection and treatment of deterioration and delayed ICU transfer. Recent systematic reviews [7–9] found moderate-strength evidence supporting the notion that implementing RRS is associated with reduced hospital cardiopulmonary arrests and hospital mortality.

Time matters when a patient deteriorates and increased time from deterioration to intervention (RRS activation and ICU transfer) has been associated with increased mortality [10], length of stay, and morbidity [10, 11]. However, several in-hospital challenges may prevent the RRS from functioning adequately [12–14]. Therefore, we must still work to understand and address barriers for timely and adequate responses in cases of patient deterioration.

When evaluating the care provided for deteriorating patients, it is also important to consider if the patient will benefit from available medical interventions or transfer to higher levels of care. Failing to make decisions regarding the limitation of medical treatment (LOMT) can lead to reduced quality of death [15]. RRS is associated with increased LOMT and end-of-life discussions [16] to prevent futile interventions in multimorbid, frail, and older patients [17]. This consideration should be built into a well-functioning RRS [18, 19].

Retrospective case record reviews, such as mortality reviews, represent a useful method for studying clinical practice. Event sequence in a deteriorating patient can be evaluated through the patient’s clinical records and charts, helping to identify quality gaps, including omission events [4, 15, 20]. Therefore, we chose this method to study deceased patient trajectories in the Department of Gastrointestinal Surgery (DGS) before and after implementing our hospital RRS.

This study aimed to investigate whether implementing and developing RRS in the DGS was associated with an overall temporal improvement and identifying needs for further improvement by studying; patient monitoring, omission event occurrences, LOMT documentation processes; unexpected death and in-hospital- and 30-day mortality rates.

## Methods

The STROBE -statement checklist for cohort studies was followed (Supplementary file 1.)

### Setting

This study was conducted in a university hospital in Norway, covering a population of approximately 400,000 inhabitants. We chose the two wards (48 beds) of the DGS where RRS implementation was initiated; as this patient group is prone to succumb due to complications from their illnesses or the surgeries performed [21, 22]. The DGS performs most types of gastrointestinal surgeries (acute and elective), from hernia and cholecystectomy to colectomy, rectal resection, pancreas, and liver surgery, but not oesophageal surgery. The intensive care capacity of the hospital is 2,2 beds / 100 000 inhabitants, which is considered to be low in an international context [23].

#### Process of RRS implementation

**Fig. 1:**
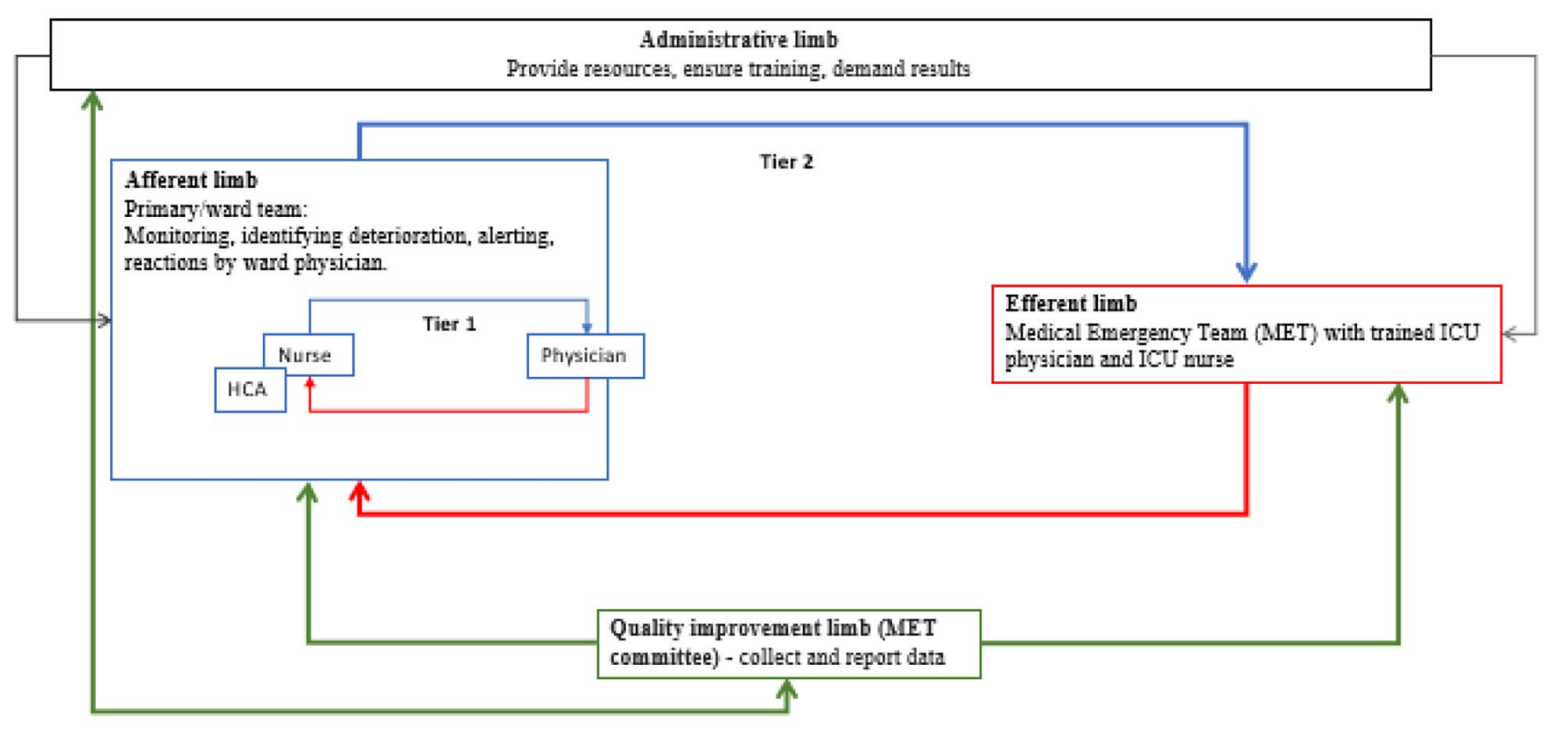
Structure of the local RRS. The limbs of the Rapid Response System. Illustrating the hospitals arrangement of the operative limbs as a two tier system. Adapted from a systematic review [14]

Before 2012, the hospital had no clearly defined procedure for vital-sign monitoring or criteria for when the nurses should alert the surgeon on duty or contact the ICU staff directly. In 2012, starting with the DGS, the study hospital implemented a two-tier RRS (Fig. 1) inspired by the RRS model at the Karolinska University Hospital, Sweden [24]. From 2014 to 2015, the system was implemented throughout the hospital. An RRS committee led the work and introduced the standard of a minimum of twice daily vital sign measurements and single-parameter Medical Emergency Team criteria (MET-c) (Supplementary file 2), which could trigger an evaluation by the Medical Emergency Team (MET). The chart for documenting vital signs was improved (Supplementary File 3). Simultaneously, there was an increase from one to two nurses in both wards for the night shift. Otherwise, there was no increase in funding.

In 2016, the RRS was further developed. An electronic observation- and medication chart (OM-chart), incorporating the NEWS (Supplementary file 3), replaced the paper-based OM-chart and the MET-c. The MET committee developed a more explicit protocol that replied to the NEWS for responses and documentation (Supplementary file 2). This response protocol included the call to decide and document all patients’ LOMT to prevent overtreatment, ensure better palliative care, and reduce unnecessary MET calls and ICU transfers. To facilitate LOMT decisions, the study wards incorporated LOMT assessments for all patients at a daily whiteboard meeting. This improvement of the RRS was carried out without increase in staff or additional funding.

### Design

**Fig. 2:**
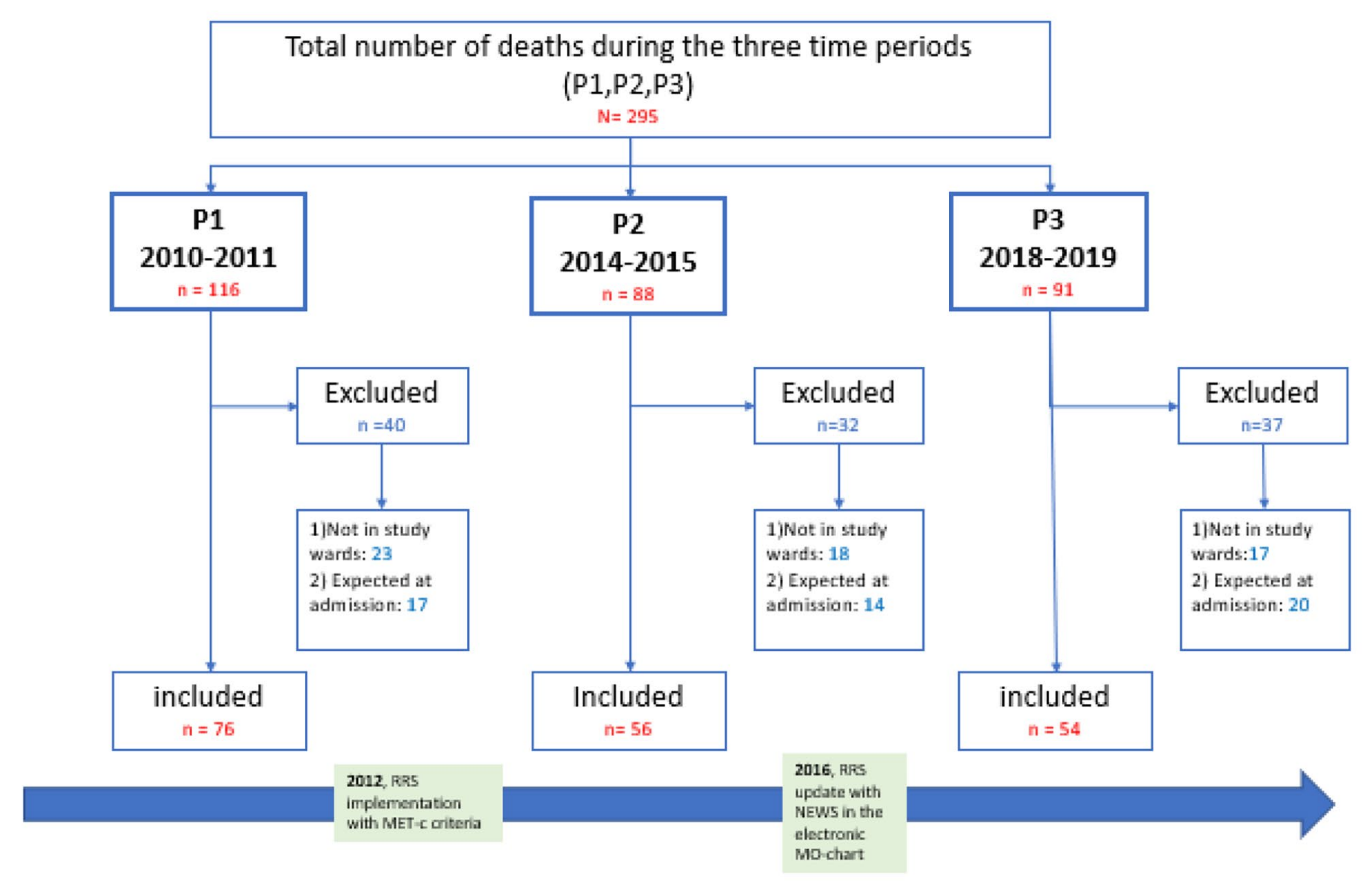
Included patients in three time periods. Overview over included and excluded patients in the three periods ( P1-P3). Illustrating when the RRS was implemented (2012) and updated (2016)

For the mortality review we chose to compare cases from three time periods Period 1 (P1), 2010/11; Period 2 (P2), 2014/15; Period 3 (P3), 2018/19). We excluded the RRS implementation period in 2012–2013 and the period of transition from single-parameter criteria to the National Early Warning Score (NEWS) in 2016/2017 (Fig. 2). For the overall mortality rates, we included all deaths in the DGS from 2010 to 2019.

### Data collection

We collected the data from two main sources; from the electronic hospital administrative- and medical records to perform a mortality review and from the regional hospital administrative data to calculate mortality rates.

#### Inclusion and exclusion criteria

For the mortality review we identified patients who died during admission to the two study wards during the three time periods (P1–P3) from the Norwegian electronic administrative and medical records system (DIPS-EPJ). Patients registered in the ward for < 2 h, were excluded. We also excluded cases from further analysis when it was evident from the admission record that all active treatments for the patient’s illness were terminated; thus, the patient was expected to succumb within a short period (Fig. 1).

To calculate mortality rates in the study wards, we included all patients registered as admitted to the study wards.

#### The mortality review process

By reviewing electronic health records and OM-charts, one of the authors (SLO) retrieved the patients’ demographic data and clinical trajectory during the hospital stay. Based on this information, an interprofessional group of reviewers, an anaesthesiologist and intensivist (ES, KS), a specialist in gastrointestinal surgery (BN), intensive care nurse (BSH), and internal medicine and emergency medicine physician (SLO) assessed the patients’ clinical pathway for omission events.

**Fig. 3:**
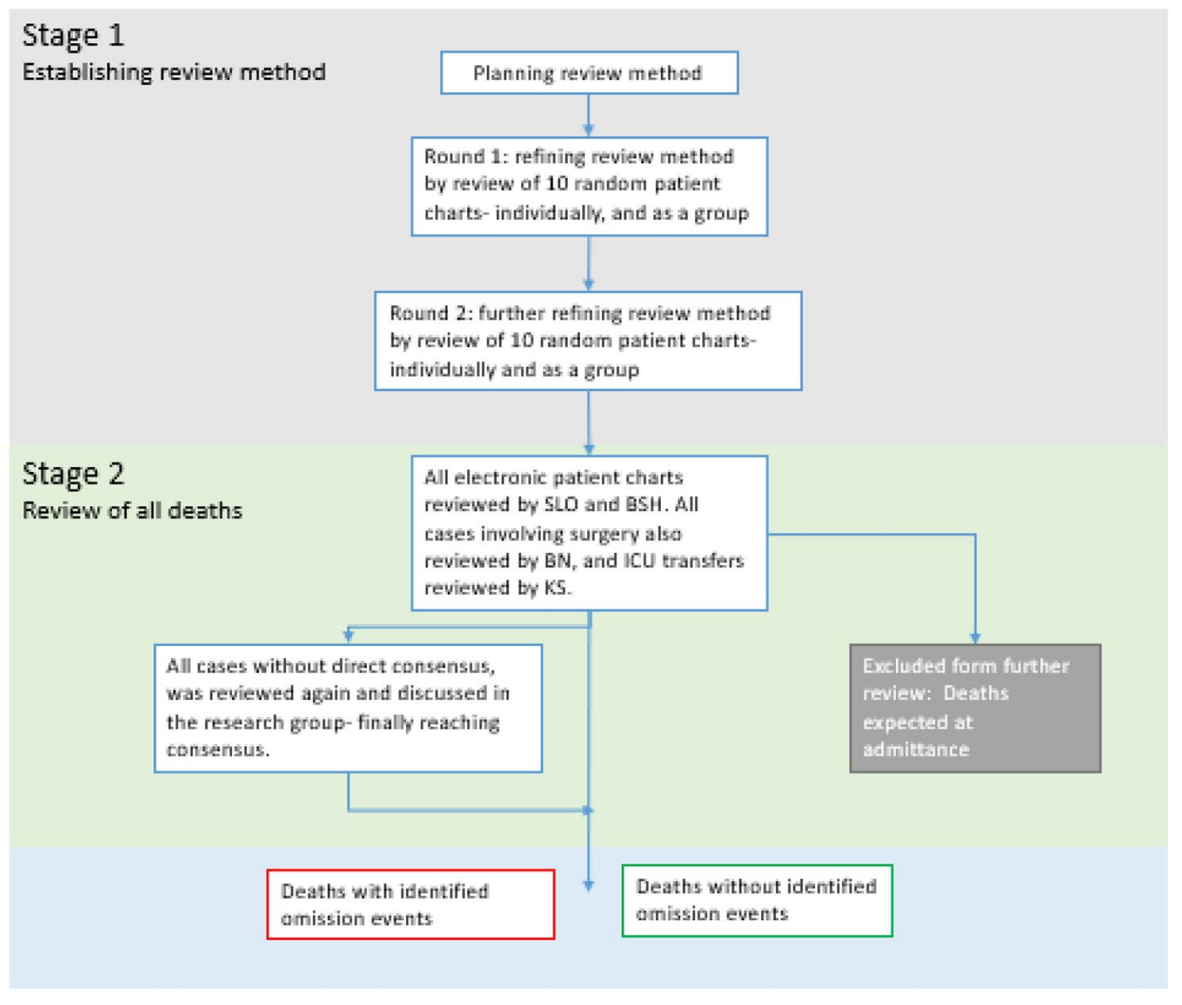
The mortality review method. Through a two-stage retrospective record review process- the research group established the review method and reviewed all included deaths, identifying cases with and without omission events

We established the inter-professional review method (Fig. 3) by conducting two pilot rounds to ensure that all reviewers were trained to evaluate the records, and in agreement when defining omission events. Two researchers (SLO and BSH) reviewed all included cases before BN, and KS reviewed selected cases (all patients undergoing surgery or being transferred to the ICU). When the group found challenging cases or had disagreements, we reviewed the case again and discussed them until a consensus was reached. The earliest records were reviewed a second time late in the process to ensure that the method did not drift during the review period.

#### Definition of omission events and unexpected death

We considered a case to be ‘failure to monitor’ when there were considerably fewer vital sign sets documented than expected when the patient was deteriorating. Further, the case was considered to be ‘failure to escalate’ when there was a clear lack of escalation from the nurse to the patient’s physician (tier 1) or a clear delay or lack of ICU consultation (MET in P2/P3) including delayed ICU transfer (tier 2). (Fig. 1) Inspired by Flaatten et al. [25], we considered deaths to be unexpected when there was no sign of deterioration in vital signs or description of deterioration in the patients’ records within 24 h before death.

#### Administrative data collection

We retrieved data for all patients admitted to the two study wards annually from 2010 to 2019 from hospital administrative data (source: Regional Information Technology partner) to study the temporal trend in the number of admittances, and in-hospital and 30-day mortality rates.

### Statistical analysis

We performed the statistical analysis using IBM SPSS statistics for Windows version 26 and R version 4.1.2 [26]. Chi-Squared tests were used to test for differences between the three time periods (P1-P3) for categorical data, with Monte Carlo simulation for data with expected cell counts less than 5. For continuous data we used the Kruskal Wallis test. We used a 5% significance level in all tests. When significant differences between periods were identified, post-hoc analysis comparing each pair of periods were done, using a Bonferroni adjustment for multiple testing. Poisson regression was used to test for temporal changes in mortality rates. (Tables 1 and 2).

**Table 1:** Comparing patient characteristics

	P1	P2	P3	p-value
**N**	76	56	54	
**Age** Median, (Q1, Q3)	77 (64, 87)	78 (72, 83)	77 (70, 84)	0.90
**Gender, male** N (%)	39 (51)	30 (54)	30 (56)	0.89
**u-CCI** Median, (Q1, Q3)	2 (0, 6)	2 (0, 4)	2 (1, 4)	0.55
**Admitted from** N (%)				
Home without care	43 (57)	32 (58)	30 (58)	0.39*
Home with care	25 (33)	11 (20)	12 (23)	
nursing home-short time	4 (5)	6 (11)	4 (8)	
Nursing home-permanent Other institution	2 (3) 1 (1)	5 (9) 1 (2)	3 (6) 3 (6)	
**Type of admittance** N (%)				
Unplanned	69 (91)	55 (98)	52 (96)	0.16*
Planned	7 (9)	1 (2)	2 (4)	
**LOS days** hospital Median (Q1, Q3)	13 (6, 22)	14 (6, 24)	11 (6, 19)	0.76
**LOS days study wards** Median, (Q1, Q3)	12 (6, 21)	13 (6, 22)	11 (6, 19)	0.81
**Number of Hospital admittances last 12 months** Median, (Q1, Q3)	1 (0, 3)	1 (0, 3)	1 (0, 2)	0.48
**Surgery performed** N (%)	31 (41)	23 (41)	18 (33)	0.93
**Reoperated one or more times N (%)**	9 (29)	4 (17)	6 (33)	0.50*

**Statistics**:

Continuous data: Kruskal-Wallis test, Categorical data: Chi-squared test. *Chi-squared test with Monte Carlo simulation.

LOS = Length of stay, u-CCI = updated Charlson Comorbidity Index

**Table 2:** **Development in patient monitoring, escalation, LOMT documentation and omission events**

	P1	P2	P3	Comparison between all periods, p-value	P1 vs. P2, p-value	P1 vs. P3, p-value	P2 vs. P3, p-value
Number of patients	**76**	**56**	**54**				
*Number of complete vital sign sets/24 h/patient. Median (Q1, Q3)	0 (0, 0)	2 (1, 2)	4 (3, 5)	**< 0.001**	**< 0.001**	**< 0.001**	**< 0.001**
*Number of simple vital signs sets/24 h/patient Median (Q1, Q3)	2 (1, 2)	2 (1, 2)	4 (3, 5)	**< 0.001**	**0.012**	**< 0.001**	**< 0.001**
**LOMT documented** N (%)	58 (76)	42 (76)	48 (89)	0.15			
**Days from admission to LOMT Median (Q1, Q3)	8 (4, 16)	8 (1,16)	3 (1, 10)	**0.011**	0.25	**0.003**	0.09
**Cardiac arrest alarms** N (%)	14 (18)	11 (20)	3 (6)	0.07			
**ICU-consult in the wards** (MET in period 2, 3) N (%)	9 (12)	17 (30)	18 (33)	**0.007**	**0.008**	**0.003**	0.738
**ICU transfer** N (%)	14 (18)	16 (29)	18 (33)	0.14			
**Cases with one or more events of omission** N (%)	30 (40)	11 (20)	6 (11)	**0.01**	**0.015**	**< 0.001**	0.216
**Types of omissions**							
Failure to monitor N (%)	20 (26)	5 (9)	1 (2)	**< 0.001**	**0.012**	**< 0.001**	0.102
Failure to escalate N (%)	14 (18)	7 (13)	2 (4)	**0.043**	0.358	**0.012**	0.092
Delayed surgery N (%)	2 (3)	2 (4)	3 (6)	0.800¤			
**Unexpected deaths** N	2 (3)	2(4)	0 (0)	0.455¤			

**Comparing groups, statistics**:

Continuous data: Kruskal-Wallis test. Categorical data: Chi-squared test. ¤Chi-squared test with Monte Carlo simulation. For the pairwise post-hoc tests, p-values < 0.0167 are considered significant due to the Bonferroni correction.

Complete vital sign set: all vital signs (pulse, O_2_ saturation, resp. frequency, BP) measured at the same period, counted the first complete 24 h stay in the ward.

Simple vital signs set: Minimum one vital sign (pulse, blood pressure, resp. frequency, O_2_ saturation) measured, counted on the first complete 24 h stay in the ward.

*Only 173 patients included, due to two cases with missing charts and 11 patients with < 24 h stay.

** of the 149 patients who had a documented LOMT.

## Results

### Mortality review

#### Patient characteristics

The socio-demographic characteristics and comorbidity [Updated Charlson Comorbidity Index (u-CCI) [27]] of the deceased patients did not differ in the three time periods, nor did the type of admittance and whether they underwent surgery during the hospital stay (Table 1).

#### Development in patient monitoring and care

RRS introduction significantly increased vital sign monitoring and documentation throughout the study time periods (Table 2). None of the patients had a complete set of vital signs in P1 because the respiratory rate was not documented. Furthermore, we found a significant increase in the number of ICU consultations after introducing MET. LOMT documentation occurred earlier during hospitalisation. We found a significant decrease in the number of patients considered to have one or more omission events during their hospital stay. The nature of the omissions changed during the study period, with fewer problems regarding monitoring and escalation. Delayed surgery and unexpected deaths were infrequent, and the number was stable during these periods. Cardiac arrest alarms trended downward during these periods without being statistically significant. There were no cardiac arrest alarms in the cases from 2019.

### Temporal trends in admissions and hospital mortality rates

**Fig. 4:**
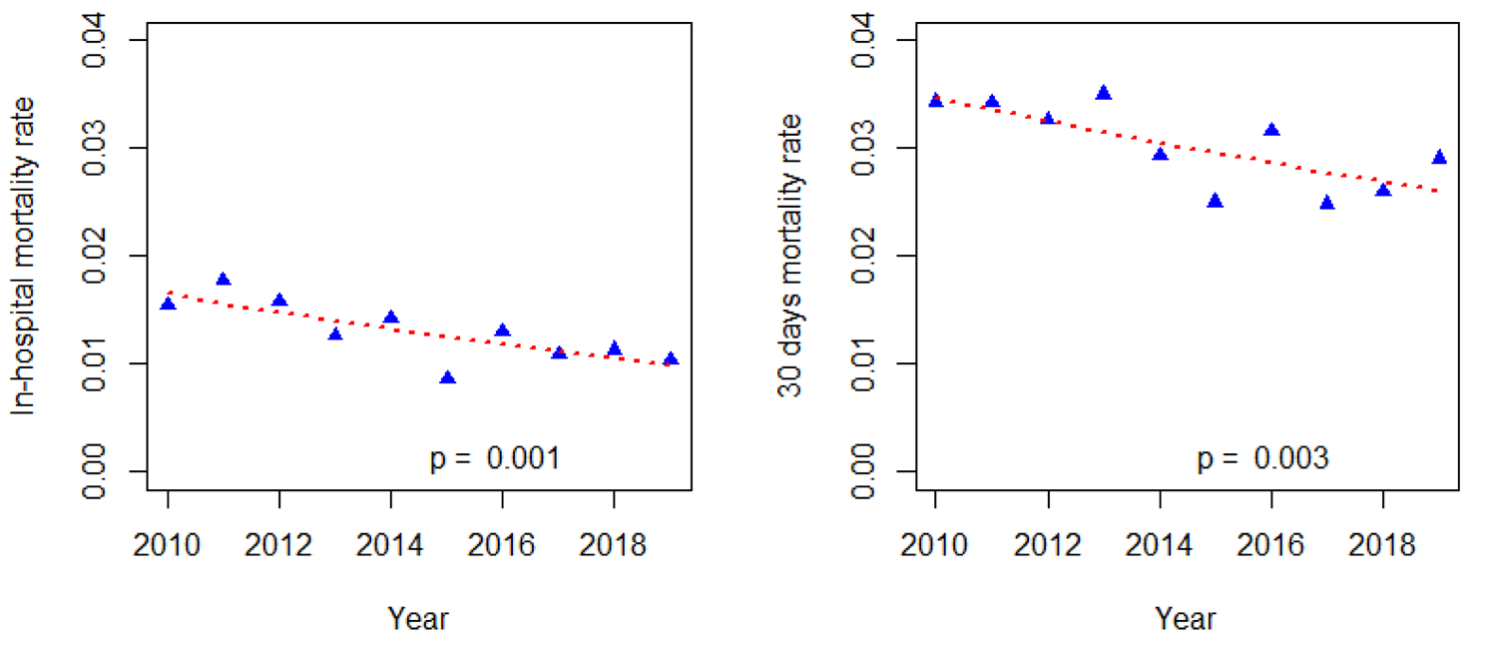
Temporal trends in in-hospital and 30-day mortality rate. In-hospital mortality rate (deaths / number of admittances): rate ratio 0,95 (95% CI 0,92 − 0,98) (P = 0.001), 30-day mortality rate (deaths within 30 days of admittance/ number of admittances): rate ratio 0.97 (95% CI 0.95- 0,99) (P = 0.003)

The total annual admittances to the two study wards increased steadily from 2,973 (2010) to 3,854 (2019). The proportion of planned admissions remained steady at approximately 30% during this period. In the same period, the in-hospital and 30-day mortality rates significantly decreased (Fig. 4). This decrease remained unchanged when adjusting for the average age of the patients.

## Discussion

### Summary of major findings

We found that implementing and further developing a university hospital RRS was associated with a temporal improvement in the ward care of patients in the DGS. There was increased systematic vital sign monitoring, earlier documented LOMT decisions, increased patient review by the ICU team, and decreased number of omission events. This was associated with a temporal decrease in the overall in-hospital- and 30-day mortality rates.

### Comparison with previous studies

We believe that establishing easily accessible and convenient systematic monitoring routines has created an important foundation for the RRS. Challenges in this fundamental limb of the RRS have been frequently reported in the literature [12, 14]. In an earlier study from this hospital, NEWS availability in the electronic OM-chart (P3) was reported to make deterioration easier to detect due to the series of time-registered measures highlighted in bright colours when vital signs deviate from normal [28]. However, ward patients are not continuously monitored; therefore, deterioration can occur between intermittent observations. International research regarding continuous vital sign monitoring outside the ICU to investigate whether this may improve patient outcomes and be cost-effective, is ongoing [29]. However, health care professionals (HCP) report worries about drawbacks, such as the potential for reduced patient contact and an increase in inappropriate escalations [30].

We found that failure to escalate seemed to decrease during the study period. We argue that this may be due to the increased availability and visibility of documented vital signs, especially during P3, and the establishment of a protocol for when to call the MET. However, this is an area for further improvement. We believe that timely escalations must be a focus for continuous attention to ensure sustainability due to many known challenges [14]. Alarm fatigue is a known challenge when monitoring patients with serious illnesses and abnormal vital signs over time [28, 31]. Furthermore, even when nurses or ward physicians recognise the deterioration, the ward-culture, and the HCPs earlier experiences of how they are treated by the MET during escalation may influence further action [14, 28]. Resources and ICU capacity are also known to influence HCP responses to patient deterioration [14, 28, 32].

The number of patients with LOMT did not change significantly during the study period, however, the LOMT was documented earlier in the patients’ hospital stay. Timely decisions on which medical interventions are suitable for a severely ill patient might prevent futile and undignified resuscitation events, prevent costly overtreatment, and make room for appropriate palliative care [33]. We speculate that earlier LOMT documentation might have influenced the lower number of cardiac arrests found during P3 and might have prevented unnecessary MET calls. In addition, through the review process, we found an opportunity to improve the quality of death further through early decision-making and LOMT documentation.

Studies on hospital mortality often use the term unexpected mortality, frequently defined as patients dying without an LOMT decision [34–36]. With our definition of unexpected deaths, we found few cases that were considered unexpected, and a considerably higher number of patients that died without a written LOMT order. Hospital implementation of processes related to decision-making and LOMT documentation is known to vary [18]. The difference found in our study, illustrates the importance of considering how unexpected death is defined. If we had considered all cases in this study with no LOMT order as unexpected, we believe this would have represented the LOMT documenting custom in the department at the time rather than actual unexpected deaths. We argue that a mortality review is a relevant method to understand whether death is unexpected, providing crucial information about patient trajectory, deterioration context, and HCP considerations.

### Implications for clinicians, hospital leaders and policy makers

To our knowledge, this is the first study on the impact of an RRS in the specific vulnerable population of gastrointestinal surgery patients. This study shows how an RRS can mature over time and gradually become more effective in its purpose, owing to continuous focus and development. We believe this study is unique in underlining how a retrospective patient record review can probe a hospital RRS, evaluate its impact, and identify strengths and quality gaps, as omissions are not readily available for statistics.

Some hospitals have established a system for performing mortality reviews closer in time to the event to search for improvement opportunities [4]. The recommended quality metrics (including measurement of cardiac arrests and predictable cardiac arrests, timeliness of response and critical care interventions and timeliness of goals of care documentation) for evaluating RRS [37] require hospitals to obtain information embedded in charts and patient records, which is not easily available. Continuous work is required to provide automatic reports on the quality metrics. Valuable reports require data to be registered and made available for a reporting system. We believe retrospective record reviews are a valuable method to probe the hospital system, as they provide detailed information about the patients’ trajectories, deterioration context, and healthcare personnel’s considerations, invaluable for knowing where to put the effort to ensure continuous improvement. Nevertheless, studying all patient records and electronic OM-charts is time consuming. To make this method a sustainable tool in daily practice, there is a need to examine how patient clinical data can be made more easily accessible.

### Strengths and limitations

Our study of hospital deaths and omission events was based on retrospective reviews of the hospital records, providing detailed information about the patients’ current hospital stay trajectories. All patients were admitted to the same two wards of gastrointestinal surgery, contributing to homogeneity of the study population. We chose to study the population of these two wards only, as they introduced the RRS at the same time ( 2012) and started the changes in 2016 at the same time. The other wards in the hospital introduced the RRS at different time intervals. The study periods for the mortality review was also limited to avoid the year of implementation of changes. This increased the comparability, but also limited the number of eligible patients. Selection bias was limited due to few exclusion criteria.

After establishing a review method, the evaluations and conclusions were performed in a broad interprofessional consensus to limit the inter-rater disagreement. Two or more researchers with different clinical backgrounds studied all cases. A statistician (JTK) was included in the planning, analysis, and reporting of study findings. However, hindsight bias and subjectivity are limitations of this study. All researchers were at the time or earlier employed at the hospital, which might have made us more positive in our judgment than an external group would have been.

Additionally, this gave us an understanding of the context, increasing the consensus credibility. None of the reviewers currently worked in the study wards. One of the reviewers currently work in another hospital. To increase generalisability, the context, including the local RRS structure, patient cohorts and the study process is thoroughly described. The transferability is for the reader to decide.

Poor documentation of the clinicians’ evaluations was evident, especially in P1, which might have influenced our conclusions. Conversely, clearer, and more complementary decision-making documentation in P3 made evaluation easier. The infrequent vital sign measures in P1, might have led us to miss cases that, in reality, should be determined as ‘failure to rescue’. In this study, it was challenging to obtain information about the context (staffing, bed occupancy, and available ICU beds). If the patient record review was performed closer in time, it would have been possible to obtain more contextual information when the response to deteriorating patients was evaluated as delayed. Improvements in the availability of diagnostic imaging and surgical methods may have contributed to decreased mortality rates.

## Conclusion

In this study, implementing and further developing an RRS led to a reduction in omission events such as failure to monitor and escalate care, earlier LOMT documentations and was associated with a temporal reduction in in-hospital and 30-day mortality rates. We found the interprofessional mortality review to be a suitable method to evaluate the RRS, providing a foundation for further improvement.

## Electronic supplementary material

Below is the link to the electronic supplementary material.

Supplementary Material 1

Supplementary Material 2

Supplementary Material 3

## Data Availability

The original data and material for the mortality review are not available due to the individual patients privacy. The administrative data sets could be available from the corresponding author on reasonable request.

## List of abbreviations

RRS: Rapid Response Systems
LOMT: Limitation of medical treatment
AE: Adverse events
ICU: Intensive care unit
MET: Medical emergency team
DGS: Department of Gastrointestinal Surgery
MET-c: Medical Emergency Team criteria
NEWS: National early warning score
OM-chart: Observation- and medication chart
DIPS-EPJ: The Norwegian electronic administrative and medical records system
P1-3: Period 1–3.
