# Supplementary material for: Reduction in omission events after implementing a Rapid Response System: a mortality review in a department of gastrointestinal surgery
Source: BMC Health Serv Res. 2023 Feb 21;23:179. doi: 10.1186/s12913-023-09159-3 (PMC9945730; doi:10.1186/s12913-023-09159-3)
Supplement: Supplementary file 2 — Supplementary Material 2 [file 12913_2023_9159_MOESM2_ESM.pptx]

## Slide 1
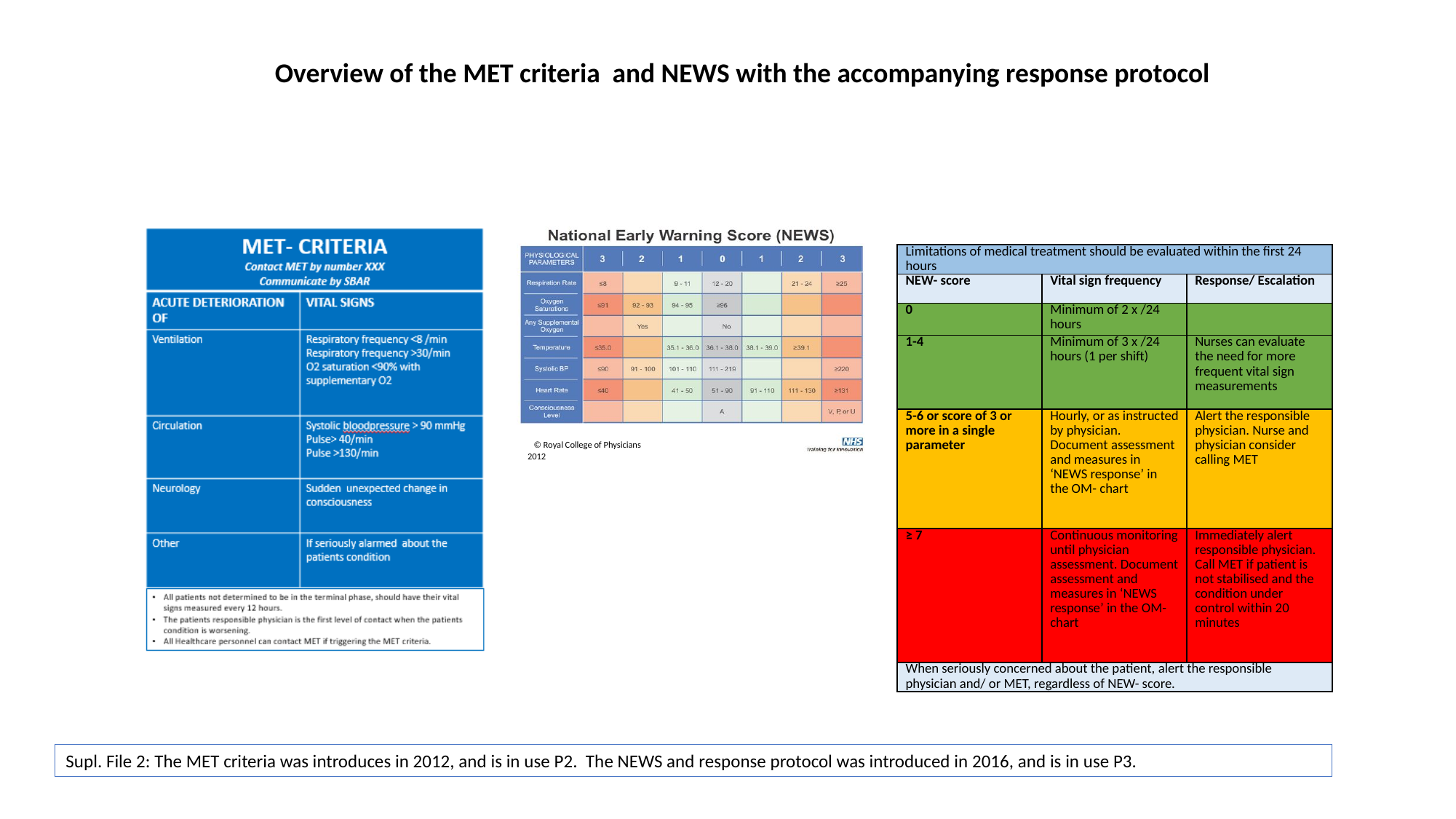

Overview of the MET criteria and NEWS with the accompanying response protocol
| Limitations of medical treatment should be evaluated within the first 24 hours | | |
| --- | --- | --- |
| NEW- score | Vital sign frequency | Response/ Escalation |
| 0 | Minimum of 2 x /24 hours | |
| 1-4 | Minimum of 3 x /24 hours (1 per shift) | Nurses can evaluate the need for more frequent vital sign measurements |
| 5-6 or score of 3 or more in a single parameter | Hourly, or as instructed by physician. Document assessment and measures in ‘NEWS response’ in the OM- chart | Alert the responsible physician. Nurse and physician consider calling MET |
| ≥ 7 | Continuous monitoring until physician assessment. Document assessment and measures in ‘NEWS response’ in the OM-chart | Immediately alert responsible physician. Call MET if patient is not stabilised and the condition under control within 20 minutes |
| When seriously concerned about the patient, alert the responsible physician and/ or MET, regardless of NEW- score. | | |
 © Royal College of Physicians 2012
Supl. File 2: The MET criteria was introduces in 2012, and is in use P2. The NEWS and response protocol was introduced in 2016, and is in use P3.
