# Supplementary material for: Reduction in omission events after implementing a Rapid Response System: a mortality review in a department of gastrointestinal surgery
Source: BMC Health Serv Res. 2023 Feb 21;23:179. doi: 10.1186/s12913-023-09159-3 (PMC9945730; doi:10.1186/s12913-023-09159-3)
Supplement: Supplementary file 3 — Supplementary Material 3 [file 12913_2023_9159_MOESM3_ESM.pptx]

## Slide 1
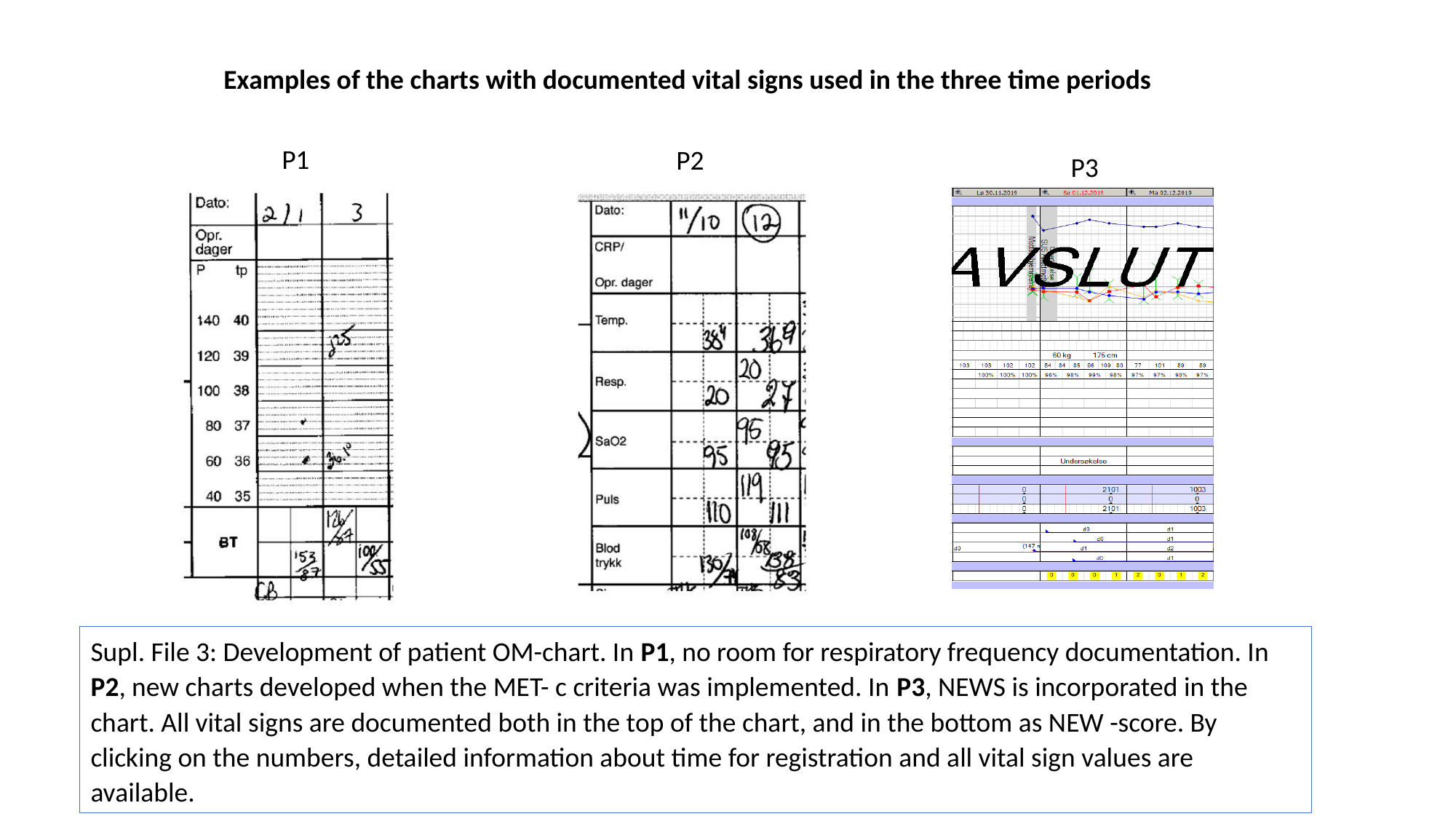

Examples of the charts with documented vital signs used in the three time periods
P1
P2
P3
Supl. File 3: Development of patient OM-chart. In P1, no room for respiratory frequency documentation. In P2, new charts developed when the MET- c criteria was implemented. In P3, NEWS is incorporated in the chart. All vital signs are documented both in the top of the chart, and in the bottom as NEW -score. By clicking on the numbers, detailed information about time for registration and all vital sign values are available.
